# The Pressure Is On – Epiphyte Water-Relations Altered Under Elevated CO_2_

**DOI:** 10.3389/fpls.2018.01758

**Published:** 2018-11-27

**Authors:** Sven Batke, Aidan Holohan, Roisin Hayden, Wieland Fricke, Amanda Sara Porter, Christiana Marie Evans-Fitz.Gerald

**Affiliations:** ^1^Department of Biology, Edge Hill University, Ormskirk, United Kingdom; ^2^School of Biology and Environmental Science, Earth Institute, University College Dublin, Dublin, Ireland; ^3^Botany Department, Trinity College Dublin, Dublin, Ireland

**Keywords:** climate change, ecophysiology, elevated CO_2_, light conditions, stomatal conductance, turgor, water-relations

## Abstract

Vascular epiphytes are a major biomass component of forests across the globe and they contribute to 9% of global vascular plant diversity. To improve our understanding of the whole-plant response of epiphytes to future climate change, we investigated for the first time both individual and combined effects of elevated CO_2_ (560 ppm) and light on the physiology and growth of two epiphyte species [*Tillandsia brachycaulos* (CAM) and *Phlebodium aureum* (C3)] grown for 272 days under controlled conditions. We found that under elevated CO_2_ the difference in water loss between the light (650 μmol m^-2^s^-1^) and shade (130 μmol m^-2^s^-1^) treatment was strongly reduced. Stomatal conductance (*g*_s_) decreased under elevated CO_2_, resulting in an approximate 40–45% reduction in water loss over a 24 h day/night period under high light and high CO_2_ conditions. Under lower light conditions water loss was reduced by approximately 20% for the CAM bromeliad under elevated CO_2_ and increased by approximately 126% for the C3 fern. Diurnal changes in leaf turgor and water loss rates correlated strong positively under ambient CO_2_ (400 ppm) and high light conditions. Future predicted increases in atmospheric CO_2_ are likely to alter plant water-relations in epiphytes, thus reducing the canopy cooling potential of epiphytes to future increases in temperature.

## Introduction

The ability of plants to modify their anatomical and physiological traits in response to their aerial and root environment, is a major adaptation to specific habitats. Extreme environments in particular have allowed plants to develop the most peculiar life-history strategies. One such group of plants are epiphytes, these plants grow on other plants (mostly trees) for physical support without extracting any nutrients directly from the host (i.e., they are non-parasitic). Most epiphytes are completely detached from the terrestrial environment and spend their entire life-cycle in the canopy. This group of mechanically dependent plants (Kelly, 1985) are also known as holo-epiphytes [see Zotz (2013) for a definition]. They can be very diverse and include many species from families within angiosperms (e.g., Orchidaceae and Bromeliaceae) and pteridophytes. Their abundance and species richness is usually highest in tropical and sub-tropical regions (Benzing, 1987; Zotz, 2016), but can also be impressive in temperate zones (Zotz, 2005). High relative air humidity (RH) and high temperatures have often been associated with the high abundance and species richness of epiphytes (Johansson, 1974; Cardelus et al., 2006; Gehrig-Downie et al., 2011; Barve et al., 2015).

Forest canopies provide a highly complex environment for epiphytes, both in terms of substrate availability (e.g., area and quality) and climate. The upper strata of forests typically experience higher levels of solar radiation and temperatures but lower RH compared to the lower forest strata (Montgomery and Chazdon, 2001; Batke and Kelly, 2014). Moreover, the air in the lower canopy is often less well mixed, causing different concentrations of atmospheric gases such as CO_2_ across the vertical forest profile (Davis et al., 2003; Stephens et al., 2007). The dynamic 3-dimensionality of forest canopies has thus allowed niche differentiation in epiphytes, likely contributing, among other factors, to the fast radiation of many epiphytic groups (Benzing, 1987, 1989; Schuettpelz and Pryer, 2009; Silvera et al., 2009; Watkins and Cardelús, 2012). The non-uniform stratification and diffusion of light (e.g., in terms of quantity and quality) is particularly striking in canopies. It is therefore not surprising that the physiological responses of epiphytes, such as photosynthesis, vary greatly within the canopy, both in response to light and VPD (vapor pressure deficit) (Stuntz and Zotz, 2001). For instance, low light or high VPD can usually cause a reduction in photosynthesis and transpiration, which results in lower biomass. Thus, sudden changes in the areal environment (e.g., as a result of moving branches or leaves) can have considerable consequences. Epiphytes are expected to respond quickly and efficiently to these brief periods of enhanced direct radiation or changes in VPD similarly to many understory species (Chazdon and Pearcy, 1991). However, under high light and water shortage, epiphytes have been shown to increase their antioxidant activity and anthocyanin content, suggesting pigments can also play an important photo-protecting function in epiphyte water-relations (González-Salvatierra et al., 2010).

In an extreme environment like the forest canopy, where plants such as epiphytes are entirely detached from the terrestrial water-source, water conservation becomes even more important to maintain optimal stomatal control. This leads to adaptations such as pseudo-bulbs in orchids, water-holding tanks in bromeliads (Benzing, 1990; Males, 2016; Zotz, 2016) or the observed prevalent crassulacean acid metabolism (CAM) in species that grow on sites where water supply is more sporadic or rare (Smith et al., 1986; Hietz and Briones, 1998). Therefore, epiphytes may have high water-use-efficiency (WUE) (Reyes-García et al., 2012) but because of this they are more constrained when responding to sudden fluctuations in their environment (Zhang et al., 2009).

It has been predicted that atmospheric carbon dioxide concentrations [CO_2_] will continue to increase in the future to levels of 463–623 ppm by the year 2050 (IPCC, 2014). In C3 species the rate of photosynthetic CO_2_ uptake is not saturated under current CO_2_ concentrations (400 ppm), which would suggest that epiphytes in particular, are likely to benefit from this increase by improving their WUE (Zotz, 2016). Stomatal closure in response to elevated CO_2_ concentrations has frequently been observed in experimental carbon dioxide enrichment studies for many non-epiphytic species (Ainsworth and Long, 2005; Ainsworth and Rogers, 2007). However, the direction (positive/negative) and the response amplitude of elevated CO_2_ on plant water loss can vary considerably (Purcell et al., 2018). Empirical data showing the effect of elevated CO_2_ on the water-relations of epiphytes is still very limited, as almost all research has primarily focused on the CO_2_ effects on changes in relative growth rate [e.g., Monteiro et al. (2009) and Zotz et al. (2010)]. Epiphytes in general are known to have notoriously slow growth rates (Schmidt et al., 2001; Zotz et al., 2001a; Schmidt and Zotz, 2002; Zotz, 2004) and also have slow assimilation rates. For example, Zotz (2016) estimated that the maximum rate of CO_2_ uptake (*A*_max_) is approximately 30% less than that of tree foliage. Collecting gas exchange data can therefore be more challenging for epiphytes, which may be the reason for the lack of data available on epiphyte responses to long-term exposure to elevated CO_2_ at high temporal resolution. Understanding the physiological response of epiphytes to fluctuations in the canopy environment and to future predicted changes in CO_2_ becomes important when trying to predict the contribution of epiphytes to forest processes.

To improve our understanding of epiphyte water-relations, the aim of this study was to test how physiological water-related traits, such as turgor and stomatal conductance (*g*_s_), respond to changes in either or a combination of elevated atmospheric CO_2_ and different light intensities. Based on evidence from other plant species (Purcell et al., 2018), the hypothesized response for epiphytes is a decrease in *g*_s_ under elevated CO_2_ and lower light levels. We assessed the expected responses at high temporal resolution using an infrared gas analyzer (IRGA) and ‘*ZIM’* turgor probes on plants that were grown under controlled conditions in growth chambers. In addition, we explore the use of the ‘*ZIM’* probe (YARA ZIM Plant Technology GmbH, Hennigsdorf, Germany) alongside IRGA measurements, to investigate whether the ‘*ZIM*’ probe could be used as a suitable tool to assess plant water-relations in epiphytes. If proven suitable, the sensor could help to overcome current methodological limitations (e.g., equipment costs) under field and controlled conditions.

## Materials and Methods

### Experimental Setup

**FIGURE 1:**
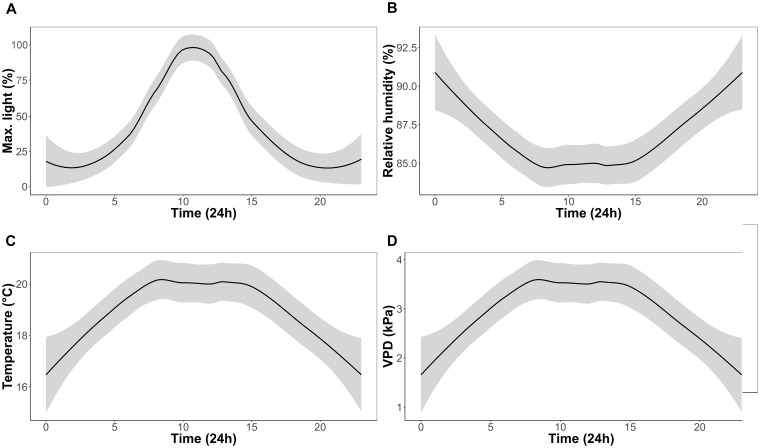
Experimental growth chamber conditions over a 24 h period for the duration of the experiment; percentage of maximum light **(A)**, relative humidity **(B)**, temperature **(C),** and vapor pressure deficit (VPD) **(D)**.

One CAM epiphytic bromeliad (*Tillandsia brachycaulos* Schltdl.) and one C3 epiphytic fern (*Phlebodium aureum* (L.) J. Sm.) were selected for this study. These species were selected as they represent an ecologically important component, in terms of their diversity, of many Central American forests (Batke et al., 2016). Specimens were provided by Bird Rock Tropicals (California, United States) and shipped to Ireland in September 2016 (Phytosanitary certificate no.: F-C-06073-05879330-7-N). Plants were initially quarantined to the greenhouse (relative humidity = 80%; temperature = 17°C; natural light) for 2 months to ensure that all individuals were healthy and pest free. Plants were transferred into four CONVIRON (Winnipeg, MB, Canada) BDR-16 plant growth chambers at the Programme for Experimental Atmospheres and Climate (PÉAC) facility at Rosemount Environmental Research Station, University College Dublin, Ireland. The chambers allowed close monitoring and control of atmospheric conditions including air temperature (*T*) (°C), relative humidity (RH) (%), light (PAR) (μmol m^-2^ s^-1^) and atmospheric O_2_ (%) and CO_2_ (ppm). For the experiment, chambers were set to a 12 h/12 h day/night cycle. Maximum day time *T* and RH was set to 20°C and 85%, respectively. Maximum night time *T* and RH was set to 17°C and 90%, respectively. Light intensity was set to reach a maximum of 650 μmol m^-2^ s^-1^ at noon (Figure 1) and O_2_ concentration was set to ambient concentrations of 21% in all chambers. A ramping program was used to ensure a uniform diurnal increase in *T*, RH and light conditions.

**FIGURE 2:**
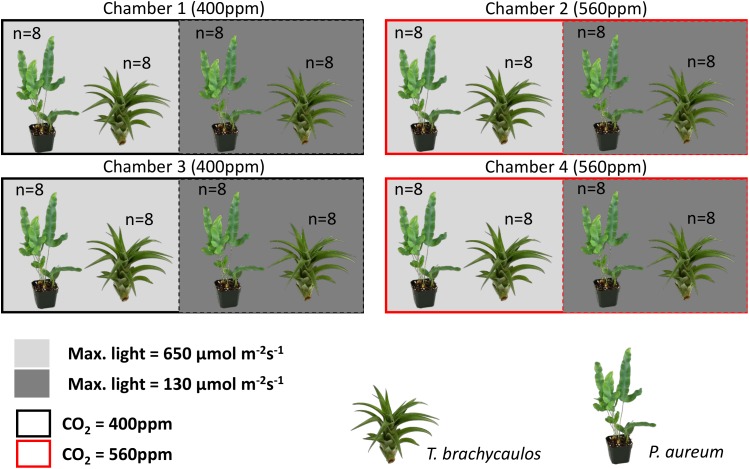
Experimental chamber design for this study. Plants in replicate chambers one and three were exposed to ambient CO_2_ conditions (400 ppm–black outline) and plants in replicate chambers two and four were exposed to 560 ppm CO_2_ conditions (red outline). Each chamber was divided by a shade net to reduce light intensity by up to 80% (see methodology for further detail). Within each treatment a total of 16 individuals per species were grown under treatment conditions for 272 days.

**FIGURE 3:**
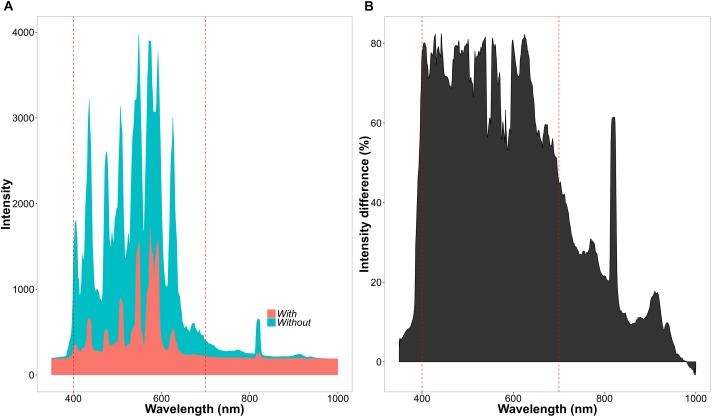
Differences of light intensity between chamber light treatments. **(A)** Light intensity across wavelengths (nm) in the light (blue = without net) and shade (red = with net) treatment. **(B)** Percentage decrease in light intensity between the light and shade treatment.

The experiment consisted of two CO_2_ treatments (with two chambers per treatment) and two light treatments within each chamber. Concentrations of CO_2_ were set to 400 ppm for the ambient and 560 ppm for the high CO_2_ treatment. Atmospheric chamber CO_2_ concentrations were monitored using a PP-Systems WMA-4 CO_2_ gas analyzer. Supplementary CO_2_ for all the chambers was provided by a compressed gas tank containing liquid CO_2_. Each chamber was divided into a light and shade treatment (Figure 2). The light treatment received chamber set-point maximum intensities of 650 μmol m^-2^s^-1^, whereas the shade treatment received maximum intensities of 130 μmol m^-2^s^-1^. The difference in light intensity between the light and shade treatment was achieved by a black 125 g m^-2^ ‘T’ shade net. The net reduced light intensity in the chamber between 60 and 80% depending on the waveband (Figure 3).

Sixteen individuals of each species per treatment were acclimatized to ambient CO_2_ (400 ppm) and light conditions (maximum 650 μmol m^-2^ s^-1^) in the chambers for 2 weeks before treatment conditions (light and CO_2_) were initiated. Starting fresh weight and maximum leaf length for *T. brachycaulos* were 33.1 g and 18 cm, respectively. For *P. aureum* all leaves were removed at the beginning of the experiment to encourage faster new growth (average plant fresh weight was 415g). Epiphytes were suspended on a metal mesh made out of chicken wire. No bark or soil medium was used. The plants were grown under treatment conditions for 272 days, in the first 3 months plants were watered daily, after which watering was reduced to three times a week. Liquid fertilizer was provided every 2 weeks (N:P:K; 18-18-18) and plants were rotated randomly within each treatment and chamber twice a month to avoid spatial acclimation (Hammer and Hopper, 1997). The water and liquid fertilizer was provided through a pressure-sprayer evenly across the plants until they were completely saturated with water (i.e., the water was spilling over from the leaf axils).

### Biomass and Leaf Area

Fresh weight (g) and size (cm) of the longest leaf of each individual bromeliad was measured at the beginning and the end of the experiment. Plant size is of particular importance in epiphytes, as many species alter their physiology during ontogeny (Zotz et al., 2001b, 2010). Leaf length was measured by determining the longest leaf from the base to the tip using a ruler. For the fern, fronds were completely removed at the beginning of the experiment and counted at the end of the experiment. Leaf dry weight was determined after drying the leaves at 40°C until the weight had stabilized. Total plant area (m^2^) was estimated by measuring the length and width of the leaf blade and multiplying it by the number of leaves per individual.

### Infrared Gas Analysis (IRGA)

**FIGURE 4:**
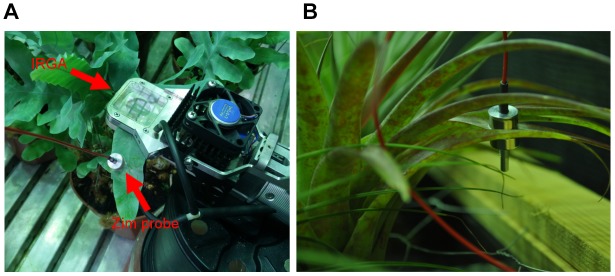
‘*ZIM’* probe sensor on a **(A)** *P. aureum* and **(B)** *T. brachycaulos* leaf. Note that the IRGA cuvette in image **(A)** is shown here for illustrative purposes only. ‘*ZIM’* and IRGA sensors were placed on separate leaves but on the same individual during data collection.

Leaf gas exchange measurements were conducted using a CIRAS-2 portable photosynthesis system and PLC (6) cuvette attachment (PP-Systems, Amesbury, MA, United States). In order to maximize the leaf area available for measurements while also reducing the amount of uncovered window space in the cuvette head a 25 mm × 7 mm head plate was used for both *T. brachycaulos* and *P. aureum* (Figure 4). The PLC (6) LED light unit was removed and all controllable conditions (RH, VPD, light, leaf, and cuvette temperature) were set to track chamber conditions. CO_2_ concentrations were maintained at 400 ppm (control) and 560 ppm (treatment) and air flow through the cuvette was constant at 200 ml min^-1^. All gas exchange measurements were taken over 24 h in a timed response program with recordings taken approximately every 40 s. Stomatal conductance was measured for six individuals per species per treatment replicate chamber using a minimum of two fully developed leaves per individual (*n* = 6 × 2 × 2). Only mature leaves were analyzed, as newly developed leaves had not reached full maturity after 8 months under treatment conditions.

### Leaf Turgor (Ψ_p_)

The ‘*ZIM*’ probe measures relative changes of turgor pressure using an artificial sensing compartment (Zimmermann et al., 2008). This is archived by clamping an intact leaf between two circular pads made up of metal magnets (Figure 4). The variable of the distance between the magnets allows adjustment of the applied magnetic force and is dependent on leaf rigidity and elasticity (Zimmermann et al., 2004, 2013). Thus turgor pressure is determined by measuring the pressure transfer through a leaf patch. Any changes in pressure transfer, for example in response to treatment conditions altering leaf water loss, can be recorded as a change in leaf, and by implication, a change in cell turgor pressure. The advantage of the ‘*ZIM*’ pressure probe is that changes in turgor can be monitored at high temporal resolution (e.g., diurnally or seasonally) and on intact transpiring plants, without having to move the device (Zimmermann et al., 2010). Turgor was monitored simultaneously on six individuals per species per treatment for 5–6 days before sensors were moved (Figure 4). The first 24 or 48h of data was discarded to ensure that the ‘settling-period’ of the sensor was not included in the final analysis (Zait et al., 2017). Measurements were repeated three times on the same plant but on different leaves.

### Leaf Solute Concentration and Anthocyanin Extraction

Leaf solute concentration was determined on two leaves per individual in each treatment using a vapor pressure osmometer (Wescor Vapro 5600). Leaves were collected, frozen in liquid nitrogen and placed into custom-made 1.5-mL centrifuge tubes, which had mesh inserts at the bottom. Tubes were stored at -80°C before thawed sampled were centrifuged at room temperature for 5 min. at 14000 rpm to extract the leaf-sap. The leaf sap collected at the bottom of the tube, and the mesh retained leaf debris. Only 10 μL of sap was required to measure the solute content (mmol kg^-1^) of samples.

Anthocyanins were extracted from two leaves per individual per chamber and processed separately. A 50 mg leaf sample from each replicate was ground in liquid N_2_ with a mortar and pestle, a solution of 1% HCI in methanol (total 150 μl) was added and the samples stored overnight in a refrigerator. The next day, 100 μl of H_2_O and 250 μl of chloroform were added. The sample was mixed well before being centrifuged for 5 min. at 5000 rpm. The absorbance of the supernatant was measured at 530nm and 657 nm using an UV-VIS spectrophotometer and the anthocyanin concentration calculated as described by González-Salvatierra et al. (2010).

### Data Analysis

Mean values of measured parameters per individual plants were used for statistical analysis. All data were tested for normality and heteroscedasticity. Only the biomass data required data transformation (log-transformation). Analysis of Variance (ANOVA) was used to test for differences between treatments. The turgor and conductance data were divided into hourly bins and the 95% confidence intervals were calculated. Linear models were used to test for the relationship between leaf turgor, *g*_s_ and transpiration. All analysis and graph plots were performed in the statistical software ‘R’ (version 3.1.2).

To calculate the total amount of water lost per species in each treatment over a 24 h period, the total area below the *g*_s_ curves (mmol/m^-2^/day) was calculated and multiplied by the mean transpiring area (m^2^) per species. Because *T. brachycaulos* is amphistomatous, the total transpiring area was multiplied by two. The molecular weight and density of water was used to convert mmol/m^-2^/day to ml/day (18g/mol, or 18 ml/mol).

## Results

**FIGURE 5:**
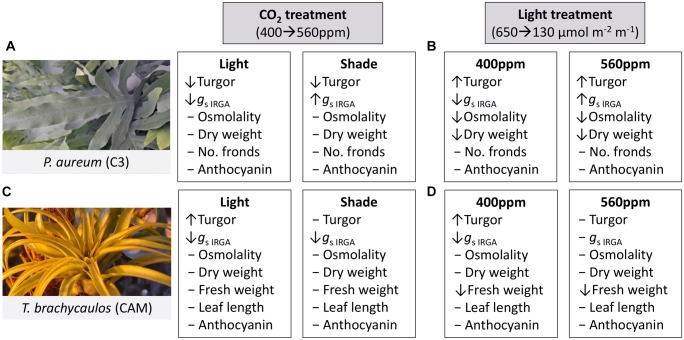
Summary of treatment effects on *P. aureum* and *T. brachycaulos* comparing the two CO_2_ and light treatments. Symbols [decrease (↓), increase (↑), no change (-)] in front of the variable names indicate the direction of response when compared at α < 0.05. **(A)** Describes the treatment effect of CO_2_ from 400 to 560 ppm (gray box) in the light (white box) and shade treatment for *P. aureum*. **(B)** Describes the treatment effect of light from 650 to 130 μmol m^-2^ m^-1^ (gray box) in the 400 ppm (white box) and 560 ppm treatment for *P. aureum*. **(C)** Describes the treatment effect of CO_2_ from 400 to 560 ppm (gray box) in the light (white box) and shade treatment for *T. brachycaulos*. **(D)** Describes the treatment effect of light from 650 to 130 μmol m^-2^ m^-1^ (gray box) in the 400 ppm (white box) and 560 ppm treatment for *T. brachycaulos*.

A qualitative summary of results is presented in Figure 5.

### Biomass

For *P. aureum* no statistically significant difference was found in the number of fronds between either, the light or CO_2_ treatments (Table 1; *t* = 0.53, *p* = 0.52). Dry weight was higher in the 560 ppm treatment compared to the 400 ppm treatment. However, this difference was not statistically significant (Table 1; *F* = 210.64, *p* = 0.35). Dry weight was higher in individuals grown under higher light compared to individuals grown in the shade treatment (Table 1; *F* = 0.89, *p* < 0.01).

**Table 1:** Mean and standard deviation (StD) of dry weight per leaf, total number of fronds, total fresh weight, and maximum leaf length.

			*P. aureum*		*T. brachycaulos*	
Variable	CO_2_ (ppm)	Light (μmol m^-2^s^-1^)	Mean	SD	Mean	SD
Dry weight per leaf/frond (g)	400	650	0.94	0.49	0.13	0.05
		130	0.35	0.26	0.11	0.03
	560	650	1.17	0.62	0.12	0.03
		130	0.46	0.27	0.12	0.05
Increase in total fresh weight (g)	400	650	nd	nd	29.28	10.43
		130	nd	nd	21.92	10.93
	560	650	nd	nd	32.75	12.61
		130	nd	nd	22.65	6.23
Increase in max. leaf length (cm)	400	650	nd	nd	2.18	1.98
		130	nd	nd	3.99	2.12
	560	650	nd	nd	1.56	1.25
		130	nd	nd	3.11	1.98
No. fronds	400	650	30.69	12.60	nd	nd
		130	32.06	11.76	nd	nd
	560	650	27.56	10.61	nd	nd
		130	29.25	11.99	nd	nd

*Individual epiphytes (*n* = 16 per treatment) were grown for 272 days under different CO_2_ and light conditions. nd, no data.*

For *T. brachycaulos*, fresh weight and maximum leaf length measured at the beginning and the end of the experiment did not differ significantly between CO_2_ treatments (Table 1; *t* = 0.54, *p* = 0.59). Overall plants increased their fresh weight by approximately 21–32 g in 272 days, with plants grown under shaded conditions showing a lower total rate of increase. Maximum leaf length increased by approximately 2–4 cm. Leaves in the shade treatment had a larger increase in leaf length compared with leaves grown in the light treatment. However, this difference was not statistically significant (*F* = 0.85, *p* = 0.36). No statistically significant differences were found in dry weight between treatments (Table 1; *F* = 0.28, *p* = 0.59).

### Turgor Measurements

**FIGURE 6:**
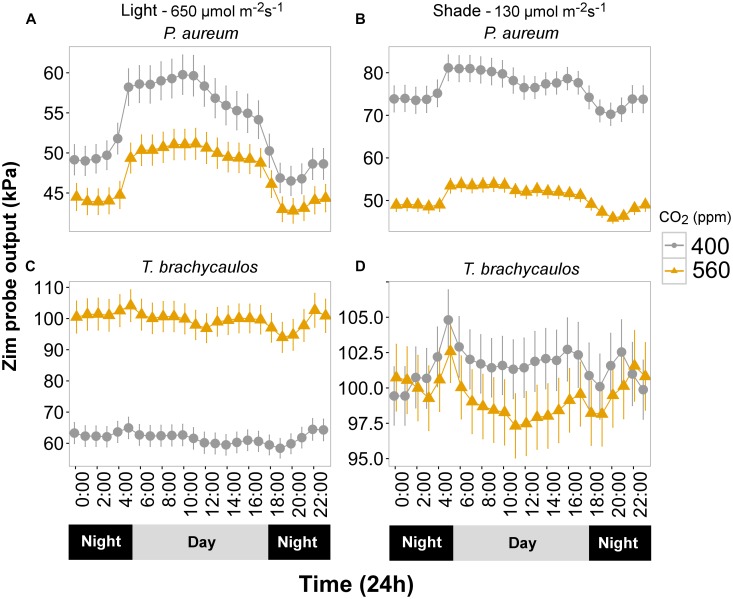
**(A,C)** Represents *P. aureum* and *T. brachycaulos* grown under high light and **(B,D)** represents *P. aureum* and *T. brachycaulos* grown under low light. Mean values with 95% confidence intervals of leaf turgor measured with the ‘*ZIM*’ probe on *P. auruem* and *T. brachycaulos* grown under experimental CO_2_ (gray circles = 400 ppm and yellow triangles = 560 ppm) and light (650 and 130 μmol m^-2^s^-1^) conditions.

Turgor (kPa) measured diurnally using the ‘*ZIM’* probe differed significantly between CO_2_ and light treatments for *P. aureum* (Figure 6). Turgor was higher in the 400 ppm CO_2_ compared with 560 ppm CO_2_ treatment. At 400 ppm turgor was higher in the shade treatment compared with the light treatment (Figure 6). This was also the case for the 560 ppm treatment, with the exception that the difference was non-significant during the night.

In contrast, turgor in *T. brachycaulos* was significantly lower at 400 ppm CO_2_ compared with turgor at 560 ppm CO_2_ in the light treatment, but no statistically significant difference in turgor was observed in the shade treatment (Figure 6). In addition, turgor did not differ between the light and shade treatment at 560 ppm but was significantly lower in the light treatment for plants measured at 400 ppm (Figure 6). The diurnal change in turgor was more prominent in *P. aureum* compared to *T. brachycaulos* and was also more well defined in the light compared to the shade treatment (Figure 6).

### Stomatal Conductance Measurements and iWUE

**FIGURE 7:**
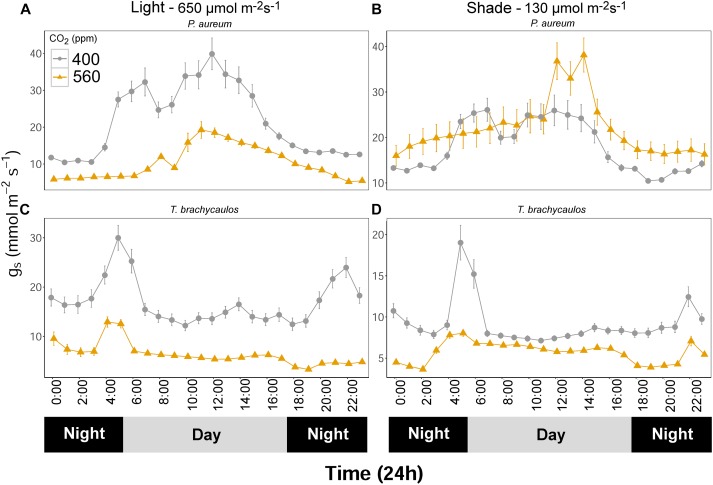
**(A,C)** Represents *P. aureum* and *T. brachycaulos* grown under high light and **(B,D)** represents *P. aureum* and *T. brachycaulos* grown under low light. Mean values with 95% confidence intervals of stomatal conductance (*g*_s_) measured for *P. auruem* and *T. brachycaulos* grown under experimental CO_2_ (gray circles = 400 ppm and yellow triangles = 560 ppm) and light (650 and 130 μmol m^-2^s^-1^) conditions.

Stomatal conductance (mmol m^-2^ s^-1^) measured using the IRGA differed between species and treatments diurnally (Figure 7). The strongest experimental effect on *g*_s_ was observed between the 400 and 560 ppm CO_2_ treatments, irrespective of the light conditions. In *P. aureum g*_s_ differed statistically between CO_2_ treatments in the light treatment. Here *g*_s_ was higher in the 400 ppm compared to the 560 ppm treatment. However, in the shade treatment the differences in *g*_s_ varied substantially between the times of the day (Figure 7B). The total amount of water loss (ml/day) for each species and treatment is summarized in Table 2. Generally, plants grown under elevated CO_2_, relative to plants growing under ambient CO_2_, had an approximate water gain of 0.1–1 ml/day/individual. For both species in the 400 ppm treatment water loss was increased in the light compared to the shade treatment (Table 2). In contrast, in the 560 ppm treatment water loss was decreased in the light compared to the shade treatment (Table 2).

**Table 2:** Mean leaf area and calculated 24 h water loss (ml/day/individual) for *P. auruem* and *T. brachycaulos* grown under experimental CO_2_ (400 and 560 ppm) and light (650 and 130 μmol m^-2^s^-1^) conditions.

Species	CO_2_	Light (μmol m^-2^s^-1^)	Leaf area (m^2^/indiv.)	Water-loss (ml/day/indiv.)
*P. aureum*	400	650	0.183	1.674
		130	0.164	0.710
	560	650	0.191	1.440
		130	0.174	1.603
*T. brachycaulos*	400	650	0.050	0.355
		130	0.051	0.134
	560	650	0.053	0.203
		130	0.058	0.137

For the bromeliad *T. brachycaulos* the measured diurnal *g*_s_ response to CO_2_ was very similar between the light and shade treatments (Figure 7). Generally, *g*_s_ was statistically significantly lower in the 560 ppm compared with 400 ppm treatment. Stomatal conductance was also higher in the light compared with the shade treatment. However, this response was less well defined in the 560 ppm treatment (Figure 7 – note the different scales).

**FIGURE 8:**
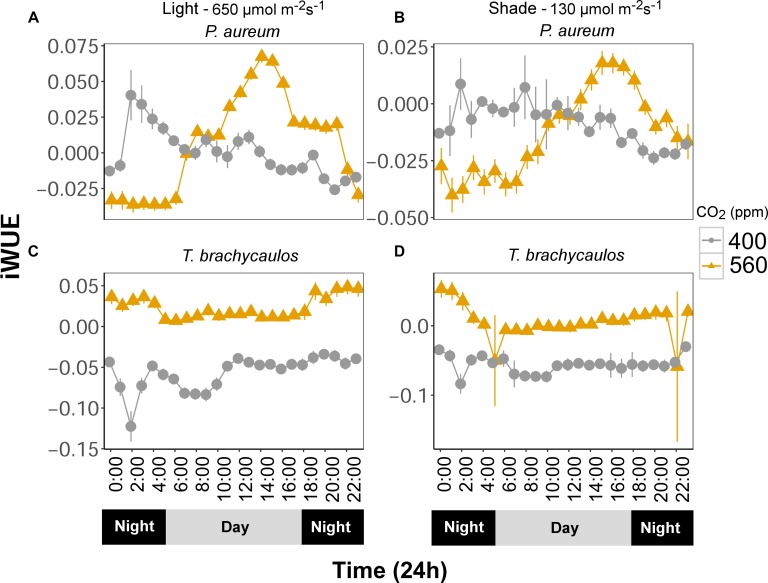
**(A,C)** Represents *P. aureum* and *T. brachycaulos* grown under high light and **(B,D)** represents *P. aureum* and *T. brachycaulos* grown under low light. Mean values with 95% confidence intervals of intrinsic water use efficiency (A/*g*_s_ = iWUE) measured for *P. auruem* and *T. brachycaulos* grown under experimental CO_2_ (gray circles = 400 ppm and yellow triangles = 560 ppm) and light (650 and 130 μmol m^-2^s^-1^) conditions.

Intrinsic water use efficiency (iWUE) changes diurnally and differed between treatments (Figure 8). Generally, iWUE was increased under elevated CO_2_. However, the increase was not always consistent throughout the day for the fern *P. aureum* (Figure 8). Both under high and low light, iWUE was highest in the early morning and afternoon for plants grown at 560 ppm (Figure 8).

The correlation coefficient between *g*_s_, transpiration and leaf turgor varied between species and treatments (Table 3). For the correlation with *g*_s_ the best fit positive correlations were observed in the light treatments compared to the shade treatments (*r*^2^ = 0.38–0.79 and -0.02–0.61, respectively). This was also the case in *P. aureum* when transpiration was correlated positively with turgor (Table 3; *r*^2^ = 0.83–0.85 and 0.57–0.7, respectively). In contrast, the positive correlations between transpiration and turgor were comparatively weak for *T. brachycaulos* (Table 3).

**Table 3:** Linear model correlations of stomatal conductance (*g*_s_) and transpiration measured with the IRGA, and leaf turgor measured with the ‘*ZIM*’ probe.

			Turgor vs. *g*_s_			Turgor vs. Transpiration		
Species	CO_2_ (ppm)	Light (μmol m^-2^s^-1^)	F	*r*^2^	*p*-value	F	*r*^2^	*p*-value
*P. aureum*	400	650	86.99	0.79	<0.01	129	0.85	<0.01
		130	37.64	0.61	<0.01	31.55	0.57	<0.01
	560	650	20.21	0.46	<0.01	112.6	0.83	<0.01
		130	10.64	0.3	<0.01	55.12	0.7	<0.01
*T. brachycaulos*	400	650	15.36	0.38	<0.01	0.48	-0.02	0.5
		130	5.91	0.18	0.02	7.46	0.22	<0.05
	560	650	18.34	0.43	<0.01	0.03	-0.05	0.87
		130	0.65	-0.02	0.43	9.91	0.28	<0.01

*F-statistic, coefficient of determination (r^2^) and p-value are given. Note that leaves on which turgor was measured differed from those where *g*_*s*_ was measured.*

### Leaf Solute Concentration and Anthocyanin Content

**FIGURE 9:**
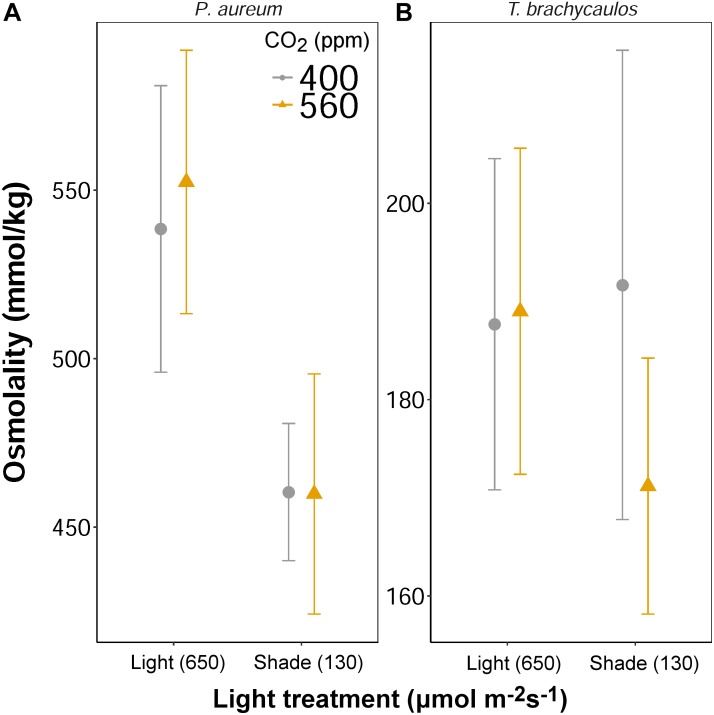
Mean values with 95% confidence intervals of leaf solute concentration measured for *P. auruem* **(A)** and *T. brachycaulos* **(B)** grown under experimental CO_2_ (gray circles = 400 ppm and yellow triangles = 560 ppm) and light (650 and 130 μmol m^-2^s^-1^) conditions.

Leaf solute concentration (mmol/kg) was determined at the end of the experiment (272 days) using a vapor pressure osmometer. No differences in solute concentration were detected for both species between the CO_2_ treatments (Figure 9). However, for *P. aureum* solutes were higher in the light compared to the shade treatment in both the 400 and 560 ppm treatment. No statistically significant difference was found as a result of light for *T. brachycaulos*. In addition, no statistically significant difference was found for total anthocyanin content (0.002–0.008 mg/g fresh weight) between any of the treatments (*F* = 0.19, *p* = 0.663).

## Discussion

The aim of the present study was to assess the impact of high CO_2_ and differences in light intensity (high and low PAR) on the water relations of two tropical epiphytes. One epiphyte was a fern (*P. aureum*), displaying C3 photosynthesis, and one a bromeliad succulent (*T. brachycaulos*), displaying CAM-type photosynthesis. We used a range of approaches to analyze diurnal changes in shoot water relations. These approaches focused on the measurement of stomatal conductance (*g*_s_) and diurnal water-loss rates, deduced from the time course of *g*_s_ and the recording of changes in turgor using the ‘*ZIM*’ probe. In addition, point analyses of leaf osmotic pressure made it possible to examine changes in leaf water potential in response to light and CO_2_ treatments.

The results show that the ‘*ZIM*’ probe provides a suitable approach to analyze changes in epiphyte water relations in the here measured species, both during a diurnal time frame and in response to changes in ambient CO_2_ and light. The results also highlight significant differences between the C3 and CAM epiphytes in our study in terms of the effect of both light and CO_2_ on water relations.

### Low Light (Shade) Significantly Impacts on the Water Loss Rate Response to High CO_2_

The effect of high CO_2_ (560 ppm) on the water loss rate per plant over a diurnal 24h period depended on the light environment of plants, to an extent that the high and low light (shade) treatments induced opposing responses in plants. In both *T. brachycaulos* and *P. auerum*, low light diminished the decreasing effect of high CO_2_ on the water loss rate of plants. Under high CO_2_ for *P. aureum*, the water loss rate increased by 126% (226% of control value). This increase was accompanied by a comparatively moderate mean increase in *g*_s_. The reason for this observation is the result of a larger area under the *g*_s_ curve at the 560 ppm treatment (Figure 7). At the same time, in *P. aureum*, *g*_s_ showed a large reduction in response to high CO_2_ in plants grown under high light, yet the plant water loss rate decreased by only 14%. In contrast, in the CAM *T. brachycaulos* grown at high light, a much higher *g*_s_ at ambient compared with high CO_2_ coincided with a much higher water loss rate under high light – yet under low-light (shade) a higher *g*_s_ under ambient compared with high CO_2_ did not coincide with any difference in water loss rate. These results allow us to make three conclusions. Firstly, water requirements for the two epiphytes studied here increase in response to high CO_2_ when plants encounter a mainly shade-dominated habitat. Secondly, our C3 and CAM epiphytes differ in their response. Third, changes in *g*_s_ cannot account for the entire change in water loss rate, the latter being also or mainly caused by changes in transpiring leaf surface and the total area under the *g*_s_ curve (the integral of *g*_s_ curves multiplied by the leaf area was used to calculate plant water loss rates).

Within a forest canopy where these epiphytes live, the capacity of individuals to utilize available light energy is very much dependent on their distribution within the forest canopy. Individuals that grow in the lower canopy are effectively shade plants, compared to individuals growing further up where the radiative force is greater. The biochemical and diffusional constraints on gas exchange such as *g*_s_ will therefore differ between the different growing sites (Gross et al., 1996). Campany et al. (2016) demonstrated in *Eucalyptus tereticornis* trees that shade leaves had lower mesophyll conductance (*g*_m_) and net leaf photosynthesis but very similar *g*_s_ compared to sun leaves. However, when they temporarily increased the light on the shade leaves, *g*_s_ increased to values that were greater than that of the sun leaves. This demonstrated that shade leaves are likely to respond quickly to sunflecks in the canopy (Woods and Turner, 1971). To increase their light interception potential (e.g., through sunflecks), shade plants often produce larger leaves. It is therefore not surprising to see that in the case of *P. aureum* water loss was greater in plants grown in shaded conditions and under elevated CO_2_, because leaf surface area increased (Table 2). In *T. brachycaulos* leaf surface area did not change much between treatments and the only large difference in water loss could be observed between the low and high CO_2_ treatment (i.e., a decrease in water loss from 400 ppm) under high light (Table 2). This decrease in water loss under elevated CO_2_ was reflected in a decrease in *g*_s_. *T. brachycaulos* is an epiphyte that is commonly found in tropical dry forests in Mexico and Central America (Mondragón et al., 2004), whereas *P. aureum* is an understory species that occurs in tropical and subtropical regions across the Americas. Under higher light and when CO_2_ concentrations are elevated (560 ppm), *T. brachycaulos* can increase WUE by increasing daily net CO_2_ uptake and by reducing *g*_s_ (Figure 8), similarly to some non-epiphytic CAM species (Drennan and Nobel, 2000). It has been shown that large numbers of epiphytes can decrease canopy temperatures (Stanton et al., 2014). However, a decrease in water loss as a result of increases in CO_2_ is likely to reduce the buffering potential of epiphytes when temperatures increase in the future (IPCC, 2014). It is therefore possible that the effect of elevated CO_2_ on epiphyte water relations could have negative feedback effects on the hosts’ response to climate change based on the two species measured.

Interestingly, the difference in *g*_s_ between CO_2_ treatments varied greatly over a 24h period in our study. In our C3 plant (*P. aureum*) the difference in *g*_s_ was highest during midday, whereas for our CAM plant (*T. brachycaulos*) the difference was greater in the early morning and late evening (Figure 7). Many CO_2_ enrichment studies do not always provide physiological information over a 24 h period (Ainsworth et al., 2008), which is particularly important for plants that are strongly driven by VPD and light intensity changes occurring in their surrounding environment. The effect of VPD on guard cell activation is often much greater in many CAM compared to C3 epiphytes (Lange and Medina, 1979; Males and Griffiths, 2017). The physiological mechanism and ecological significance of differences in diurnal rhythms between C3 and CAM plants has been greatly discussed in the literature (Males and Griffiths, 2017). Stomatal conductance in C3 plants has been shown to increase in the morning and reaches maximum values around noon (Males and Griffiths, 2017), while in CAM plants *g*_s_ is highest at night (Phase I) and then decreases to minimum values around noon (Phase III). In addition, light and VPD are more influential on the diurnal patterns of C3 compared to CAM plants (Szarek and Ting, 1975; Dodd et al., 2002). In our study, *g*_s_ in both the C3 fern (*P. aureum*) and CAM bromeliad (*T. brachycaulos*) conformed largely to their predicted diurnal rhythms (Figure 7). However, under elevated CO_2_ concentrations the amplitude of the diurnal pattern in *g*_s_ was less well defined and is likely the result of increased stomatal closure.

### ‘ZIM’ Probe Suitable to Assess Water Relations of Epiphytes

The ZIM probe was used to record changes in turgor, rather than to attempt the measurement of absolute values of turgor, which requires several assumptions (Zimmermann et al., 2008). For this reason, values obtained for the same species grown under different conditions can be compared more on a qualitative rather than quantitative basis. All the correlations between turgor and *g*_s_ were positive (Table 3) within species and treatments.

The changes in turgor in response to treatments were generally in agreement with predicted changes in turgor if one assumes that a higher water loss rate due to increased *g*_s_ should lead to a reduced water supply to cells and turgor in these cells. However, this does not necessarily mean that turgor will decrease. Turgor only decreases if the rate of water loss of the cell increases more than that of water uptake into that cell. Scaled up to the leaf level, that could mean that if *g*_s_ and leaf water loss increase, turgor could also increase if the rate of water import into the leaf increased even more. Reversely, if the solute accumulation in cells or the rate of water delivery from root/non-shoot tissue to leaf cells increases, this could allow stomata to open more and *g*_s_ to go up because of higher turgor. The sequence of these events are difficult to determine and raised the question whether stomata are the controlling component. In *T. brachycaulos* grown under high-light, a higher *g*_s_ and water loss rate at 400 ppm compared with 560 ppm CO_2_ coincided with a lower turgor; at low-light, the difference in *g*_s_ between CO_2_ treatments was comparatively small, and there was hardly any difference in turgor. Similarly, in *P. aureum* grown under low-light, a higher *g*_s_ and plant water loss rate was accompanied by a lower turgor at high compared with low CO_2_. The one treatment where changes in turgor did not match changes in *g*_s_ and plant water loss rate was *P. aureum* plants grown at high-light. Here, a higher *g*_s_ and slightly increased plant water loss rate were accompanied by a higher, not lower turgor. This apparently contradictory result could be explained by an increased rate of water import into leaves. We conclude from the above that the ZIM probe provides a close approximation of changes in *g*_s_ and plant water loss rates under most, though not all, treatment x species combinations tested, as changes in solute concentrations inside the leaf and/or changes in the leaf wall tissue and mechanical properties can also be important in regulating water transfer within a plant (Cutler et al., 1977).

Measuring physiological traits of epiphytes in the field is notoriously difficult. Access to individuals for example can be problematic, particularly when heavy infrared gas analyzer (IRGA) equipment needs to be employed to measure several species across multiple canopy layers. The ‘*ZIM*’ probe used in our study can potentially be used as a proxy to measure physiological epiphyte responses under different growing conditions (Zimmermann et al., 2013; Zait et al., 2017). We found that for *P. aureum* there were strong positive correlations between leaf turgor measured with the ‘*ZIM*’ probe and transpiration measured with the IRGA (*r*^2^ = 0.6–0.9) in both the 400 and 560 ppm treatment (Table 3). Similarly, *g*_s_ correlated moderately with leaf turgor in both CO_2_ treatments for *T. brachycaulos* (*r*^2^ = 0.3–0.8). The strength of the correlations was significantly poorer when plants were measured under shaded conditions (Table 3). This is not surprising, as turgor is likely to be more impacted by the larger changes in water loss through stomata under higher light compared to the marginal water loss under shaded conditions. In addition, the correlations are stronger in the C3 fern compared to the CAM bromeliad. The lower *g*_s_ rates observed in the CAM bromeliad are a common adaptation of many epiphyte species (Zotz and Winter, 1994) that are adapted to water deficient environments, thereby ensuring that tissue desiccation occurs slowly, whilst maintaining cell turgor. Although the osmotic potential in many epiphytes is high (Martin et al., 2004), their low rates of transpiration and higher water storage capacity often makes epiphytes very drought tolerant. Many epiphytes such as bromeliads often have angled leaf-shapes, making it currently not possible to use the ‘*ZIM*’ probe on these species, particularly when they are juveniles. Yet, on many larger epiphytes which have a more regular leaf surface and in which water loss is mostly regulated by active stomatal responses, the ‘*ZIM*’ probe could be a valuable proxy, as it is a cheaper and more versatile sensor for measuring diurnal changes in epiphyte water loss.

### Leaf Water Potential Responds to High CO_2_ in the C3 but Not CAM Epiphyte

Leaf water potential is calculated as the difference between leaf turgor and leaf osmotic pressure, both being defined at cell level (Fricke, 1997). Qualitative changes in turgor were recorded with the ZIM probe, whereas leaf osmotic pressure was quantified using a VAPRO osmometer. We do not know how much of a difference in ‘*ZIM*’ probe output between any two treatments amounts to any particular mmol/kg-change in leaf osmotic pressure. Therefore, we cannot calculate changes in leaf water potential, yet we can ascertain with only some uncertainty whether leaf water potential changed in response to treatments. In the CAM *T. brachycaulos* grown under high light, both leaf osmolality and leaf turgor did not differ between CO_2_ treatments. Leaf water potential will have stayed the same or changed only little. Similarly, in *P. aureum* grown under low-light, both leaf osmolality and leaf turgor were slightly lower at 560 ppm compared with the 400 ppm CO_2_ treatment. Again, leaf water potential will have changed little. In contrast, in the C3 epiphyte *P. aureum* grown under high CO_2_ and low and high, both the leaf turgor and leaf osmolality data points toward a decrease leaf water potential. The CAM epiphyte displayed an isohydric, whereas the C3 epiphyte displayed an anisohydric response to the high CO_2_ treatment. Furthermore, changes in *g*_s_ were not necessarily in line with changes in leaf water potential. For example, a much higher *g*_s_ at 400 ppm compared with 560 ppm in *P. aureum* coincided with no change in leaf water potential. Despite a lower *g*_s_ at 560 ppm compared with 400 ppm CO_2_, *P. aureum* plants grown under high-light and 400 ppm CO_2_ exhibited a decrease in leaf water potential compared to plants grown at 560 ppm CO_2_.

Enrichment studies have previously reported increases in leaf thickness in C3 plants under elevated CO_2_ (Thomas and Harvey, 1983). Increased cell wall thickness means that the leaf becomes firmer and thus has a higher Young’s modulus (Ding et al., 2014). Increased turgor results in increased cell volume (Steudle et al., 1977), but proportionally more force is required to maintain the same amount of pressure (Matthews et al., 1984). It is therefore likely that in the case of *P. aureum* possibly under low light, changes in cell wall structure affected absolute turgor values more under elevated CO_2_ concentrations.

### Epiphyte Growth Rates

The few studies that have investigated the effect of elevated CO_2_ on epiphytes have primarily focused on its effect on relative changes in growth rate (Li et al., 2002; Croonenborghs et al., 2009; Monteiro et al., 2009; Zotz et al., 2010; Wagner and Zotz, 2018). For example, Monteiro et al. (2009) showed that relative growth rate was only increased by 6% for different epiphytes grown under elevated CO_2_ (from 280 to 560 ppm). However, when light and nutrients were increased, epiphyte growth was stimulated by 21% and 10%, respectively. A more recent study by Wagner and Zotz (2018) showed that relative growth rate was increased in two epiphytes by approximately 35 and 60% under elevated CO_2_ (from 400 to 800 ppm). We found no significant change in growth under elevated CO_2_ (from 400 to 560 ppm) in our study. However, similar to previous studies, the increase in growth of the two epiphytes was stimulated by higher levels of light (∼35–55% increase in growth). It is likely that differences between our study and others is a result of the duration of the experiment. Our study was conducted over 272 days, which is significantly longer than other CO_2_ experiments on epiphytes.

## Conclusion

We demonstrated that our two epiphytes respond similarly physiologically to other plant species (both C3 and CAM) by closing their stomata and thus reducing water loss under elevated CO_2_. In addition, under elevated CO_2_ water loss in the light and shade treatment was strongly reduced compared to the ambient CO_2_ treatment. Given the importance of epiphytes to forest primary productivity [e.g., 13% of the total forest net primary productivity in a forest in Costa Rica (Richardson et al., 2000)], future predicted increases in atmospheric CO_2_ (IPCC, 2014) is likely to increase relative growth rate (Wagner and Zotz, 2018) and reduce water transfer from epiphytes (this study). A reduction in water loss by epiphytes is likely to negatively affect the host plants ecophysiology and forest ecosystem processes, by reducing the cooling potential of epiphytes (Stanton et al., 2014) in response to higher predicted temperatures and lower VPD in the future.

## Author Contributions

SB designed and carried out the experiments, analyzed the data, and wrote the manuscript. AH helped with the IRGA data collection and assisted SB with the development of the experiments. RH, AP, and CE-F helped with the project set-up and data collection. WF assisted in the measurement of osmotic pressure and data analysis. All authors provided critical feedback and helped to shape the research, analysis, and manuscript.

## Conflict of Interest Statement

The authors declare that the research was conducted in the absence of any commercial or financial relationships that could be construed as a potential conflict of interest.
